# A Hybrid Path-Oriented Code Assignment CDMA-Based MAC Protocol for Underwater Acoustic Sensor Networks

**DOI:** 10.3390/s131115006

**Published:** 2013-11-04

**Authors:** Huifang Chen, Guangyu Fan, Lei Xie, Jun-Hong Cui

**Affiliations:** 1 Department of Information Science & Electronic Engineering, Zhejiang University, Hangzhou 310027, China; E-Mail: chenhf@zju.edu.cn; 2 School of Electronic Information, Shanghai Dianji University, Shanghai 200240, China; E-Mail: fgyright@126.com; 3 Department of Computer Science & Engineering, University of Connecticut, Storrs, CT 06029, USA; E-Mail: jcui@engr.uconn.edu

**Keywords:** underwater acoustic sensor networks (UWASNs), path-oriented code assignment (POCA), POCA-CDMA-MAC, round-robin, funneling effect

## Abstract

Due to the characteristics of underwater acoustic channel, media access control (MAC) protocols designed for underwater acoustic sensor networks (UWASNs) are quite different from those for terrestrial wireless sensor networks. Moreover, in a sink-oriented network with event information generation in a sensor field and message forwarding to the sink hop-by-hop, the sensors near the sink have to transmit more packets than those far from the sink, and then a funneling effect occurs, which leads to packet congestion, collisions and losses, especially in UWASNs with long propagation delays. An improved CDMA-based MAC protocol, named path-oriented code assignment (POCA) CDMA MAC (POCA-CDMA-MAC), is proposed for UWASNs in this paper. In the proposed MAC protocol, both the round-robin method and CDMA technology are adopted to make the sink receive packets from multiple paths simultaneously. Since the number of paths for information gathering is much less than that of nodes, the length of the spreading code used in the POCA-CDMA-MAC protocol is shorter greatly than that used in the CDMA-based protocols with transmitter-oriented code assignment (TOCA) or receiver-oriented code assignment (ROCA). Simulation results show that the proposed POCA-CDMA-MAC protocol achieves a higher network throughput and a lower end-to-end delay compared to other CDMA-based MAC protocols.

## 1. Introduction

Since underwater acoustic sensor networks (UWASNs) are useful for many oceanic applications, such as the oceanic environment monitoring, oceanic geographic data collection, offshore exploration, assisted navigation, disaster prevention and tactical surveillance, more and more researchers have being paying attention to this topic in recent years. However, the channel of UWASNs has many specific characteristics, such as long propagation delay, low available bandwidth and large Doppler spreading, many technologies for UWASNs are, therefore, different from those used in terrestrial wireless networks [1].

The propagation delay of the underwater acoustic channel is five orders of magnitude higher than that in terrestrial radio channel. The available bandwidth determined by both transmission range and frequency usually has a limit of 40 km·kbps [2]. In UWASNs, designing a suitable MAC protocol is an important and challenging issue due to the specific channel characteristics [1–3]. Considerable effort has been invested and many MAC protocols were recently proposed for UWASNs.

Due to their simplicity, schedule-based protocols are widely used for UWASNs. In [4], a spatial-temporal MAC (ST-MAC) scheduling for UWASNs based on time division multiple access (TDMA) is proposed. In this protocol, a spatial-temporal conflict graph and the vertex coloring method are used to deal with the spatial-temporal uncertainty. In [5], an ordered carrier sense multiple access (ordered CSMA) based on the round-robin scheduling for single-hop UWASNs is proposed. In this protocol, each node distributed with a circular order of transmission keeps sensing the carrier and transmits data following its previous node in the transmission order. Moreover, the time synchronization and long waiting time are not needed. In [6], a schedule-based protocol, UMAN-MAC, is proposed for UWASNs. In the protocol, a sender broadcasts its own schedule in order for other nodes to go to sleep and wake up at the proper time, so this protocol is energy-efficient. However, this protocol only works well in networks where the nodes transmit data periodically.

Contention-based protocols have also been widely investigated in UWASNs. In [7], a tone-based contention protocol, T-Lohi, is proposed for single-hop UWASNs. For exploiting space-time uncertainty and long latency to detect collisions and count contenders, the protocol becomes a flexible, fair and stable MAC. In [8], an energy-efficient MAC protocol is proposed, where senders transmit their packets at a suitable time with notification of collisions by the intended receiver.

In multiple access collision avoidance (MACA) [9], small size control packets, request-to-send (RTS) and clear-to-send (CTS), are used for collision avoidance on the shared channel. When a node has a data packet to send, it first transmits an RTS packet to request the channel. The intended receiver replies with a CTS packet if it receives RTS correctly. With correct CTS, the sender transmits its data packet(s). However, hidden/exposed terminal problems are not fully resolved by the MACA protocol.

Moreover, protocols based on the three-way handshake mechanism are also widely used in UWASNs. Distance aware collision avoidance protocol (DACAP) [10] is a contention-based protocol with the three-way handshake mechanism. According to the long propagation delay, DACAP makes a sender wait for a mandatory waiting time before it sends the data packet after receiving CTS; in addition, it allows the destination to send a warning packet to the source to cancel the transmission if it receives an RTS packet from another node. An adaptive propagation delay tolerant collision avoidance protocol (APCAP) is proposed in [11]. In this protocol, a sender is allowed to respond to other senders while waiting for the CTS packet from an intended receiver, and this modified mechanism improves the network throughput. In order to alleviate the funneling effect [12] happening in a localized and sink-oriented network, a funneling MAC for UWASNs (FMAC-U) is proposed in [13]. With the three-way handshake mechanism, this protocol makes the sink receive data packets from multiple neighboring nodes in a fixed order during each round of handshakes.

COPE-MAC [14] is another protocol based on a three-way handshake mechanism. It uses parallel reservation and carrier sensing methods to avoid the packet collision when a node has received more than one request to send data. Focusing on the case in which two nodes can transmit to each other at around the same time without collision, a bidirectional-concurrent MAC protocol (BiC-MAC) is proposed in [15]. Since the sender-receiver pair is allowed to transmit data packets to each other in a successful handshake, the BiC-MAC protocol greatly improves the data transmission efficiency and channel utilization.

The triple hidden terminal problem in single-transceiver multi-channel long propagation delay underwater networks, the multi-hop, multi-channel and long-delay hidden terminal problem are defined in [16]. Then, a cooperative underwater multi-channel MAC protocol based on a three-way handshake mechanism and a cooperative collision detection mechanism is proposed to solve the triple hidden terminal problem.

Slotted FAMA [17] is a contention-based protocol based on floor acquisition multiple accesses (FAMA) [18] for UWASNs. In this protocol, all nodes share the common slot synchronization, and initiate the RTS-CTS handshake at the beginning of a slot. Compared to TDMA, Slotted FAMA has no idle slots.

On the other hand, receiver-reservation-based MAC protocols have been investigated to avoid the hidden terminal problem. In [19], authors proposed the receiver-initiated packet train (RIPT) protocol for multi-hop UWASNs. In the RIPT protocol, an intended receiver invites senders to transmit the data packets, and coordinates data packets from multiple neighboring nodes in a packet chain manner.

In order to take advantage of the low delay of contention-based MAC protocols and the high throughput of schedule-based MAC protocols, designing a hybrid MAC protocol has also been investigated for UWASNs. CDMA is the most promising technique used in a hybrid MAC protocol since it is robust to frequency-selective fading and it can easily compensate for the effect of multi-path transmission at the receiver. In [20], authors proposed a distributed protocol for long latency access networks (PLAN), in which a node uses a unique spreading code to encode its signals (such as RTS, CTS and DATA) before transmitting. Then, the intended receivers broadcast a CTS packet for several accumulated RTS packets and receive data packets from multiple senders at the same time. In [21], by combining ALOHA and CDMA, a transmitter-based CDMA MAC protocol is proposed for UWASNs. Since a closed-loop distributed algorithm is used to set the optimal transmit power and the code length to minimize the near-far effect [22], the protocol achieves a low channel access delay, low energy consumption and high network throughput. Inspired by the theory of compressed sensing, a distributed energy-efficient sensor network scheme, random access compressed sensing (RACS), is proposed in [23]. This protocol is suitable for long-term deployment underwater networks in which energy saving is of crucial importance. It also prolongs network lifetime since a simple and distributed scheme is used to eliminate the need of scheduling. In [24], a hybrid spatial reuse TDMA (HSR-TDMA) protocol based on time division technology and direct sequence spread spectrum technology is proposed for broadcasting UWASNs. This protocol improves the network performance since the near-far problem is resolved.

The MAC protocols mentioned above are proposed to reduce the packet collision or deal with the long propagation delay problems in UWASNs, and the network throughput is improved to some extent with a random topology. However, many applications of UWASNs have some special characteristics which should be considered in designing the MAC protocol. For example, in a UWASN for oceanic environment monitoring and oceanic data collection, the network generally consists of a sink and many sensor nodes deployed surrounding the sink. That is, the network topology is sink-oriented, in which sink is the destination of the information generated at sensor nodes all over the network. When data packets are transmitted hop-by-hop from the sensor nodes to the sink, the funneling effect occurs [12], but the existing MAC protocols mentioned above fail to deal with the funneling effect in UWASNs. In [13], FMAC-U is proposed to improve three-way handshake mechanism between the sink and one hop neighboring nodes in order to alleviate the funneling effect. However, this protocol did not consider the funneling effect problem in multi-hop networks.

In order to resolve the funneling effect in multi-hop UWASNs, an improved CDMA-based MAC protocol, named path-oriented code assignment (POCA)-CDMA-MAC protocol, is proposed in this paper. In the proposed protocol, sensor nodes perform a round-robin scheduling in the same route and CDMA technology for different routes. That is, the nodes in the same path are assigned the same spreading sequence, and then they transmit data packets pre-processed with the spreading sequence in a round-robin method. With POCA-CDMA-MAC protocol, a sink can receive data packets from multiple paths simultaneously, and the packet collision, therefore, is reduced. Since the number of paths is much less than that of nodes, the length of the spreading sequences required in POCA-CDMA-MAC protocol is shortened. Hence, the proposed POCA-CDMA-MAC protocol performs well, in term of a high network throughput, a low packet loss rate and a small end-to-end delay.

The remainder of the paper is organized as follows: in Section 2, we introduce the system model and formulate the problem we aim to resolve. In Section 3, the POCA-CDMA-MAC protocol for UWASNs is presented. In Section 4, the proposed MAC protocol is evaluated by simulations. In Section 5 further insights into the proposed MAC protocol are provided. Section 6 gives the conclusions and suggestions for future work.

## 2. System Model and Problem Formulation

**Figure 1.**
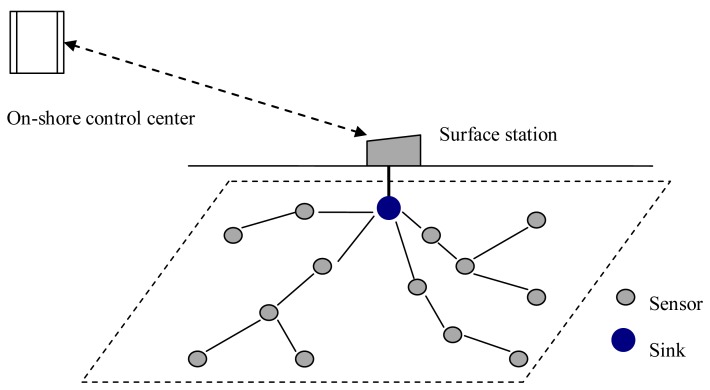
Network architecture.

A typical architecture of a UWASN is shown in Figure 1. A sink is located at the center of the monitored area, many sensor nodes are deployed surrounding the sink, and a surface station is deployed to act as a gateway between the on-shore control center and the sink. The information gathered by sensor nodes in the monitored field transmits to the sink hop-by-hop. The data packets at the sink are forwarded to the surface station via cable. Finally, the data packets are transmitted to the on-shore control center by radio signals [25,26].

**Figure 2.**
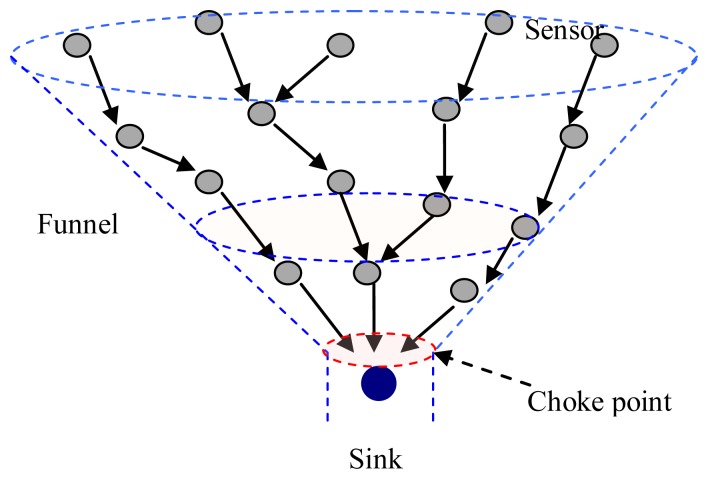
Funneling effect.

In UWASNs, the sink is assumed to be a sufficient energy supply and capable of handling parallel communications with senor nodes, all sensor nodes are homogenous and quasi-stationary. In Figure 1, since the data packets generated at sensor nodes are transmitted to the sink hop-by-hop in a many-to-one pattern, the funneling effect happens, as shown in Figure 2. From the Figure 2, we observe that the number of sensor nodes near the sink is much less than that of sensor nodes far from the sink. Therefore, the sensor nodes near the sink need to transmit more data packets than those far from the sink and the quantity of data transmitted by the neighboring nodes of the sink is the most, and the area around the sink becomes the choke point for the whole network.

**Figure 3.**
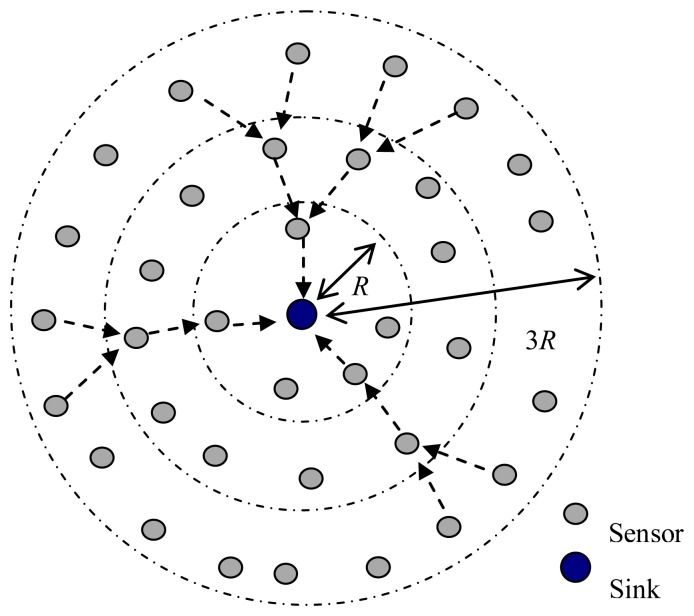
A network with uniformly distributed sensor nodes.

As shown in Figure 3, the sensor nodes are randomly deployed in a circular area. When the distance from the sink to the network edge is larger than the maximum transmission range of sensor nodes, the sensor nodes near the sink should act as the intermediate nodes to forward the data from sensor nodes located far away from the sink.

Let the maximum transmission range of sensor nodes be *R* km, the sensor nodes are uniformly deployed with the average density of *ρ*, and data packets in each node arrive in a Poisson process with the arrival rate *λ*.

The number of sensor nodes in the *i* th circle is *N_i_* = *ρA_i_*, where *A_i_* is the area of the *i*th circle, and *A_i_* = (2*i* −1)*πR*^2^. The data packets generated within *T* seconds by the sensor nodes in the *i*th circle are *n*_G_, *_i_* = *λTN_i_*. When the distance from the sink to the edge of the network is *d*_max_ = *MR*, the data packets forwarded by the sensor nodes in the *i*th circle are:

(1) $$n_{\text{F},i}=\lambda T\overset{M}{\underset{j=i+1}{\text{∑}}}N_{j}$$

The average data packets transmitted by each sensor node in the *i* th circle, including the data generated by itself and the forwarded data, are:

(2) $$n_{i}=\frac{n_{\text{G},i}+n_{\text{F},i}}{N_{i}}=\frac{\lambda T\overset{M}{\underset{j=i}{\text{∑}}}\left(2j-1\right)}{2i-1}$$

**Figure 4.**
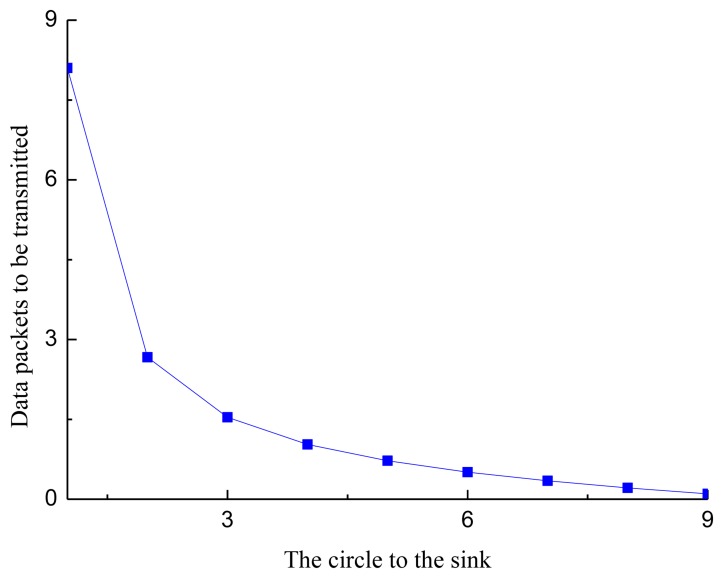
The average number of data packets transsmitted by each sensor node.

Figure 4 shows the number of data packets transmitted by each sensor node in a circle, where *M* = 9 and *λT* =1. From Figure 4, we observe that the data transmitted by the sensor nodes near the sink are much more that of the sensor nodes far from the sink. The reason for this phenomenon is that the sensor nodes near the sink need to forward the data from the sensor nodes far from the sink.

The fairness index of sensor nodes transmitting data packets, the Jain's fairness index, is defined as:

(3) $$FI=\frac{{\left(\overset{M}{\underset{i=1}{\text{∑}}}n_{i}\right)}^{2}}{M\overset{M}{\underset{i=1}{\text{∑}}}n_{i}^{2}}$$

**Figure 5.**
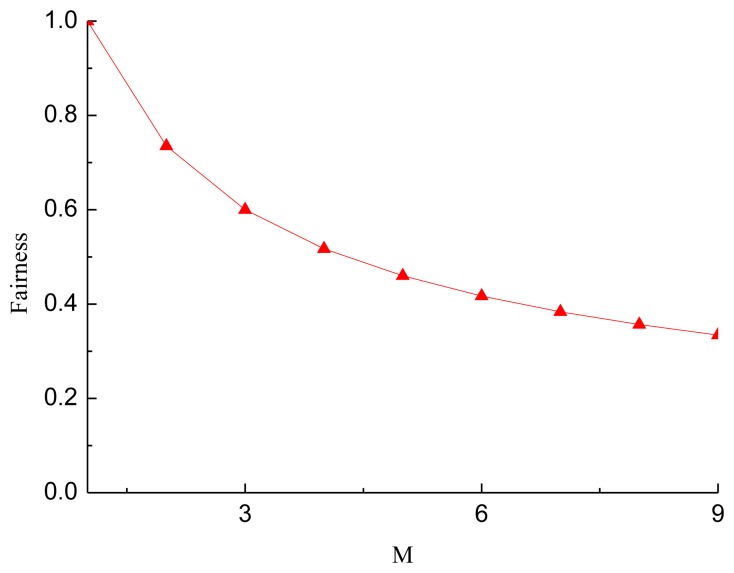
Fairness of sensor nodes in each circle transmitting data with different network radius.

Figure 5 shows the fairness of sensor nodes in each circle transmitting data with different network radius. From Figure 5, we observed that when the radius of the network equals the maximum transmission range of the sensor nodes, data packets transmitted by sensor nodes are almost the same, and then the fairness is 1. This is the case of a single-hop network, as sensor nodes only need to transmit their own data to the sink. When the radius of the network increases, the fairness decreases. The reason for this phenomenon is that the data packets forwarded by sensor nodes near the sink increases as the network radius increases. When *M* = 9, the fairness is 0.308, the worst.

As analyzed above, in UWASNs the traffic intensity increases as data packets move more closely toward the sink. Hence, the funneling effect leads to not only the increase of packet collision, network congestion and packet loss, but also the increase of the energy consumption of nodes near the sink.

Because of the long propagation delay and low available bandwidth, it is necessary to alleviate the funneling effect in designing the MAC protocol for UWASNs. Although the throughput is improved by mitigating the hidden terminal problem and reducing the packet collision to some extent, existing MAC protocols for UWASNs do not consider the funneling effect in a multi-hop UWASN. The RTS-CTS handshaking and the CDMA-based method in PLAN make receivers concurrently receive packets from multiple sources, which is a good choice for alleviating the funneling effect. However, since the protocol considered that each node is allocated a quite long spreading sequence using distributed TOCA, its efficiency is low. The T-Lohi and the Ordered CSMA protocols without the handshake mechanism work well in single-hop UWASNs, but they cannot achieve good performance in multi-hop networks. Furthermore, the funneling-MAC is proposed to resolve the funneling effect problem with a hybrid TDMA/CSMA approach in terrestrial wireless sensor networks. However, due to the long propagation delay in UWASNs, a slot time in TDMA protocol, the sum of the transmission delay of data packets and the maximum propagation delay of the network, is also quite long, and the performance of networks, therefore, is reduced.

## 3. Proposed POCA-CDMA-MAC Protocol

In this section, an improved CDMA-based protocol for UWASNs, named POCA-CDMA-MAC, is presented. A round-robin method and CDMA technology are utilized to reduce the packet collisions. It allows the sink to receive packets from multiple neighbors belonging different paths at the same time. It also works well in a multi-hop network with the hidden/exposed terminal problems.

### 3.1. Overview of POCA-CDMA-MAC Protocol

In the proposed POCA-CDMA-MAC protocol, the sink determines the routes and spreading sequences for nodes. When sensor nodes are deployed, routes will be built by the sink in the initialization phase, the detail of the determination of the routes is shown in Section 3.2. Each node is ordered with its position in the path. That is, the first node in the path is the first sender, and the next node in the path is the second sender, and so on. The sink is the destination of all paths. Each node follows the order to transmit in a round-robin manner. When a node except the first sender wants to transmit a data packet, it has to wait for data packets from its previous node. The start transmitting time of the first node at each path is determined by the sink, which is described in the Section 3.3.

**Figure 6.**
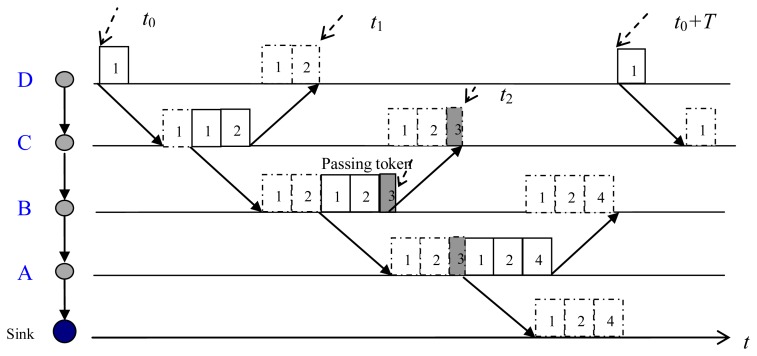
Data packet transmission via an established path.

The packet transmission via an established path is demonstrated in Figure 6. At the start transmitting time, *t*_0_, the first sender, node D, sends its data packet (packet 1) to its next-hop node, node C. Receiving packet 1 from node D, node C first forwards packet 1, and then transmits its own packet (packet 2). After transmitted packet 1, node D does not delete the packet until it receives the forwarded packet from node C. When node B received and forwarded packets from node C (packets 1 and 2), it only sends a token (packet 3) since it has no generated data. The token is used to trigger next transmission. Receiving packets from node B, node A forwards packets (packets 1–3) and transmits its own packet (packet 4) to the sink. Then, node D starts another transmission at *t*_0_ + *T*.

### 3.2. Path-Oriented Code Assignment (POCA)

When several packets from one-hop neighbors arrive at the sink at the same time, packet collision happens. To avoid the collision, each one-hop neighbor of the sink pre-processes packets with a spreading sequence. Unlike the TOCA and the ROCA, the sink uses the path-oriented code assignment (POCA) mechanism. Nodes in the same path are assigned with a same spreading sequence, whereas nodes in different paths are assigned with different spreading sequences.

In the initialization phase, the sink assigns a pseudo-random binary spreading sequence to each established path in the network. It is assumed that the spreading sequence is orthogonal or quasi-orthogonal. In general, since the number of the established paths is much less than the number of nodes, the length of the spreading code used in POCA is much shorter than that of TOCA or ROCA.

**Figure 7.**
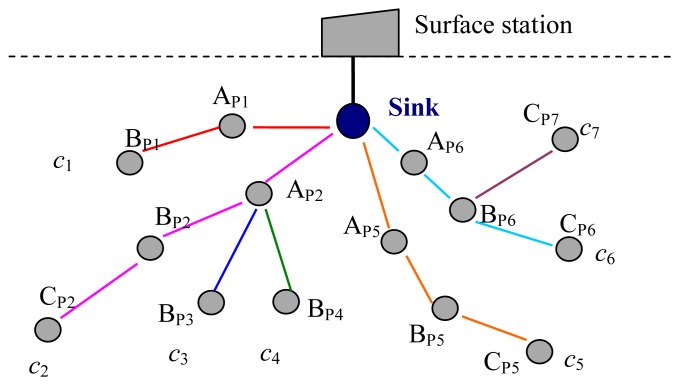
Spreading sequences assignment for paths.

As shown in Figure 7, there are a sink and some sensor nodes belonging to seven paths around the sink in the network. There are two nodes, A_P1_ and B_P1_, in path 1. There are three nodes, A_P2_, B_P2_ and C_P2_, in path 2. There are two nodes, A_P2_ and B_P3_, in path 3. There are two nodes, A_P2_ and B_P4_, in path 4. There are three nodes in paths 5–7. A_P2_ is shared by three paths, paths 2–4. A_P6_ and B_P6_ are shared by two paths, paths 6 and 7.

In the initial phase, the sink generates and sends spreading sequences to its neighbor nodes according to the number of established paths in the network. When A_P1_ received its spreading sequence (*c*_1_) from the sink, it transfers *c*_1_ to B_P1_. The sink sends three spreading sequences (*c*_2_, *c*_3_, *c*_4_) to A_P2_, and then A_P2_ assigns these three spreading sequences to B_P2_, B_P3_, and B_P4_. When B_P2_ has received *c*_2_, it transfers *c*_2_ to C_P2_. Other nodes in the paths 5–7 are assigned the spreading sequences in the same way. Finally, seven spreading sequences are assigned to seven paths separately.

When B_P1_ has packets to send, it pre-processes its packets with *c*_1_ and transmits the processed packets to A_P1_. When A_P1_ received the packets from B_P1_, it forwards them to the sink, and pre-processes its own packets with *c*_1_, and transmits them to the sink. When A_P2_ received the packets from B_P2_, B_P3_ and B_P4_, it identifies them with different spreading sequences and forwards them to the sink. Then it pre-processes its own packets with *c*_2_ and transmits them to the sink.

As mentioned above, there are two cases about the transmission time of each node in a given path. In one case, for a node, such as node A_P1_ as shown in Figure 6, there is only one previous node. In this case, A_P1_ forwards the packets received from its previous node, B_P1_, and pre-processes its own packets with its spreading sequence, *c*_1_, and transmits them. In another case, for a node, such as A_P2_ as shown in Figure 6, there is more than one previous node, three previous nodes, nodes B_P2_, B_P3_ and B_P4_, for A_P2_. In this case, A_P2_ forwards the data packets from its three previous nodes after received and identified these packets with their spreading sequences. Then, A_P2_ pre-processes its own packets with its own spreading sequence, *c*_2_, and transmits them.

When the sink has received the data packets from different paths with different spreading sequences, it decodes the packets with corresponding spreading sequences. For example, B_P1_ pre-processes packet *DATA*_BP1_ with *c*_1_ and transmits it. Receiving pre-processed packet, $\text{DAT}{{\text{A}}^{\prime }}_{{\text{B}}_{\text{P}1}}$, the sink extracts *DATA*_BP1_ with the same spreading sequence, *c*_1_, as shown in Equations (4) and (5):

(4) $$\text{DAT}{{\text{A}}^{\prime }}_{{\text{B}}_{\text{P}1}}=\text{DAT}A_{{\text{B}}_{\text{P}1}}⊕c_{1}$$

(5) $$\text{DAT}A_{{\text{B}}_{\text{P}1}}=\text{DAT}{A^{\prime }}_{{\text{B}}_{\text{P}1}}⊕c_{1}$$

In a real UWASN, there are some nodes except the sink are shared by more than one path, and the number of the path is more than the number of one-hop neighbors of the sink. Hence, the spreading sequences can be also reused in two paths when the distance between two paths is more than two times of the maximum transmission range of nodes. From Figure 7, there are seven paths in the network, but only four one-hop neighbors of the sink. Hence, code *c*_7_ can be the same as one of the codes *c*_1_–*c*_4_.

### 3.3. Timing of the First Node Sending Packets

The timing of the first transmission node in each path is an important issue. If the first node starts transmitting at the proper time, nodes may share the channel well, and this favors reducing the packet collisions and improving the network throughput.

The time for the first node to send packets depends on the traffic load of the network, the deployment of nodes and the hops of the path, and so on. For simplicity, the first node sends its packets periodically, and the period interval of each path is assigned by the sink according to the hops of the path, the maximum data packets transmitted by a node each time, and the maximum propagation delay between two neighboring nodes. A long periodic interval may be set for a path with many nodes and high traffic load, while a short periodic interval may be set for a path with few nodes and low traffic load.

In the POCA-CDMA-MAC protocol with a round-robin method, the first node at a path starts the transmission during a periodic interval, and the sink has to wait for long time to receive DATA packets from its neighbors. In a path with more than two hops from the first node to the sink, the first node can start the transmission when the sink is receiving DATA packets from its neighbors. As shown in Figure 6, node A can start another transmission at *t*_2_, and *t*_2_ < *t*_0_ + *T*. Hence, the sink waits for a short time to receive DATA packets, which increases the network throughput.

### 3.4. Analysis of the Maximum Network Throughput

The throughput is an essential metric to evaluate the performance of MAC protocols. In sink-oriented networks, the throughput is defined as the ratio of the total received packets by the sink to the packets it can receive during a given time [19]. When the spreading sequence technology is not used in the system, the sink can only receive packets from a neighboring node. For simplicity, a network with only one path is considered, and the network throughput is the same as the throughput of the path. Hence, the network throughput can be written as:

(6) $$S=\frac{L\cdot n_{\text{DATA},0}}{r\cdot T}$$

where, *S* is the network throughput, *L* is the length of each DATA packet, *r* is the data rate of the system. And *n*_DATA,0_ is the number of DATA packets received by the sink during *T*.

From Figure 6, there are a sink and four nodes at the path. When each node transmits its DATA packets on its order, and the first node transmits its DATA packets within *T*, the maximum throughput of network with only one path is:

(7) $$S_{\max}=\frac{L\cdot n_{\text{DATA},1}}{L\overset{\text{Hop}}{\underset{i=1}{\text{∑}}}n_{\text{DATA},i}+r\cdot \overset{\text{Hop}}{\underset{i=1}{\text{∑}}}D_{\text{P},i}}$$

where, *S*_max_ is the maximum network throughput. *n*_DATA,1_ is the number of DATA packets transmitted by a one-hop neighbor (node A) each time, including forwarded DATA packets. *n*_DATA_,*_i_* is the number of DATA packets transmitted by the *i*th hop neighbor each time, *Hop* is the number of hops at a path, and *Hop* = 4 in Figure 6. And *D*_p_,*_i_* is the propagation delay of packets transmitted from the *i*th hop neighbor to the (*i*-1)th hop neighbor. In schedule-based networks without packet collision, the sink can receive all of the DATA packets transmitted by its one-hop neighbor (node A), then *n*_DATA,0_ = *n*_DATA,1_.

Since the CDMA technology is used in the system, the data packets are pre-processed with the spreading sequence before transmission, and the sink can receive from multiple neighboring nodes simultaneously. On the other hand, the transmission delay of the packets is expanded. Hence, the maximum throughput in a path can be re-written as:

(8) $$S_{\max}=\frac{\eta \cdot L\cdot n_{\text{DATA},1}}{L_{\text{c}}\cdot L\overset{\text{Hop}}{\underset{i=1}{\text{∑}}}n_{\text{DATA},i}+r\overset{\text{Hop}}{\underset{i=1}{\text{∑}}}D_{\text{P},i}}$$

where, *L*_c_ is the length of the spreading sequence, *η*, the factor of the CDMA technology, is relevant to the parameters of the technology, such as the signal-to-interference-plus-noise ratio (SINR), the spreading gain, the power of the receiving signal and the length of the spreading sequence. Since the paths are set up and the spreading sequences are assigned statically by the sink in the initialization phase, each node has relative static neighboring nodes. The interference signals from other senders can be neglected if the sink assigns a long spreading sequence to nodes.

When many paths are set up around the sink, the nodes at each path will transmit the DATA packets pre-processed by different spreading sequences. Then, the maximum network throughput is:

(9) $$S_{\max}=\overset{N_{\text{path}}}{\underset{k=1}{\text{∑}}}S_{\max,k}$$

where *S*_max_,*_k_* is the maximum throughput of the *k* th path, and can be obtained in Equation (8)*N*_path_ is the number of the paths in the network.

In a sink-oriented UWASN, the sink can build the paths with the same hops. When each node at a path transmits the same number of data packets, the number of one-hop neighbors, *n*_DATA,1_, of these paths is the same, the maximum network throughput, therefore, can be achieved. That is:

(10) $$S_{\max}=\frac{\eta \cdot L\cdot n_{\text{DATA},1}\cdot N_{\text{path}}}{L_{\text{c}}\cdot L\overset{\text{Hop}}{\underset{i=1}{\text{∑}}}n_{\text{DATA},i}+r\overset{\text{Hop}}{\underset{i=1}{\text{∑}}}D_{\text{P},i}}$$

**Figure 8.**
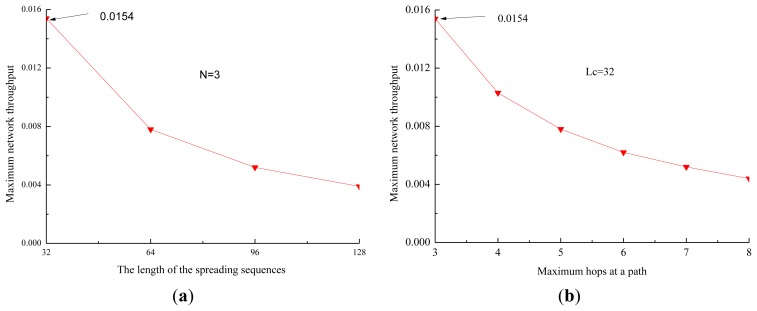
The impact of the length of the spreading sequences and the number of hops at a path on the maximum network throughput of the proposed protocol. (**a**) The length of the spreading sequences; (**b**) The number of hops at a path.

When there are eight one-hop neighbors in the network, the data rate is 1 kbps, and the length of the data packets is 4,000 bits, the propagation delay is 4 s. It is also assumed that each node in a path transmits packets with the same size. The impact of the length of the spreading sequences and the hops of the path on the maximum network throughput of the proposed protocol is shown in Figure 8.

From Figure 8a, we observe that the maximum network throughput decreases quickly when the length of the spreading sequences increases. The reason is that the length of the data being transmitted increases quickly as the length of the spreading sequences increases, and the efficiency of the channel decreases fast, and then the maximum network throughput decreases. From Figure 8b, we observe that the maximum network throughput decreases quickly when the number of hops at a path increases. The reason is that the time of the channel used by one-hop neighbors decreases when the number of hops increases, and then the efficiency of the channel and the maximum network throughput decrease.

Therefore, two useful conclusions can be drawn. First, the network throughput increases when the number of DATA packets transmitted by one-hop neighbors during *T* increases, and it increases along with the ratio of the number of DATA packets transmitted by one-hop neighbors of the sink to the number of DATA packets transmitted by other nodes. Second, the performance of the network throughput is improved by alleviating the effect of the long propagation.

## 4. Simulation Results

To evaluate the performance of our proposed POCA-CDMA-MAC protocol, simulations were performed under different traffic loads. The performance of the proposed MAC protocol, in terms of the network throughput, the packet drop rate and the end-to-end delay, is compared to two MAC protocols for UWASNs, the Slotted FAMA in [17] and the RIPT protocol in [19]. The proposed MAC protocol with different spreading sequences for each path and the proposed MAC protocol with spreading sequences reuse for different paths, are labeled as “proposed MAC without spatial reuse” and “proposed MAC with spatial reuse”.

The packet drop rate is the ratio of the number of packets dropped by all nodes to the number of packets generated by them. The end-to-end delay is the average time of the packets received from generation to reception. That is:

(11) $$\text{Packet drop rate}=\frac{n_{\text{d}}}{n_{\text{G}}}$$

(12) $$\text{End}-\text{to}-\text{end delay}=\frac{\overset{n_{\text{DATA},0}}{\underset{k=1}{\text{∑}}}\left(t_{\text{R},k}-t_{\text{G},k}\right)}{n_{\text{DATA},0}}$$

where, *n*_d_ is the number of packets dropped by all sensor nodes, *n*_G_ is the number of packets generated by all sensor nodes. *t*_R_,*_k_* is the time of the *k*th packet received by the sink, *t*_G_,*_k_* is the time of the *k*th packet generated by one of sensor nodes.

**Figure 9.**
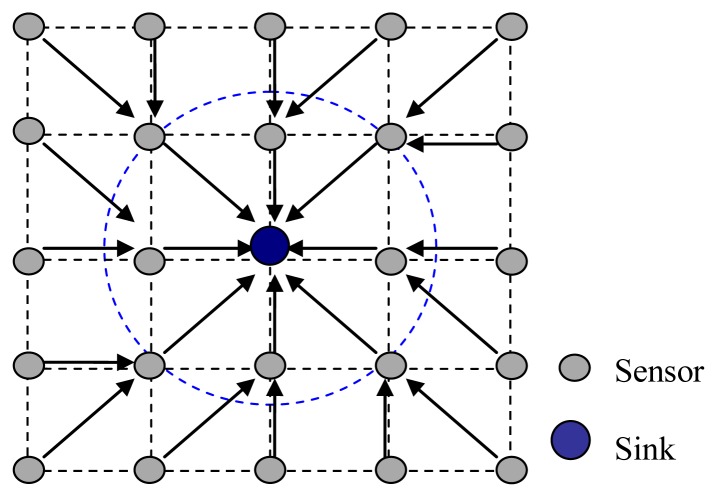
Network topology in simulations.

The network topology used for simulations is shown in Figure 9. There are 24 sensor nodes deployed in a grid topology, and a sink is located at the center. Instead of precisely placing each node at the grid intersection point, some randomness is introduced by allowing each node to deviate from the grid intersection point within 10% of the grid spacing in both x and y directions. In the network, the grid spacing is 5 km, and the transmission range is set to be 1.75 times of the grid spacing so that each sensor node has exactly eight neighbors in its transmission range.

**Figure 10.**
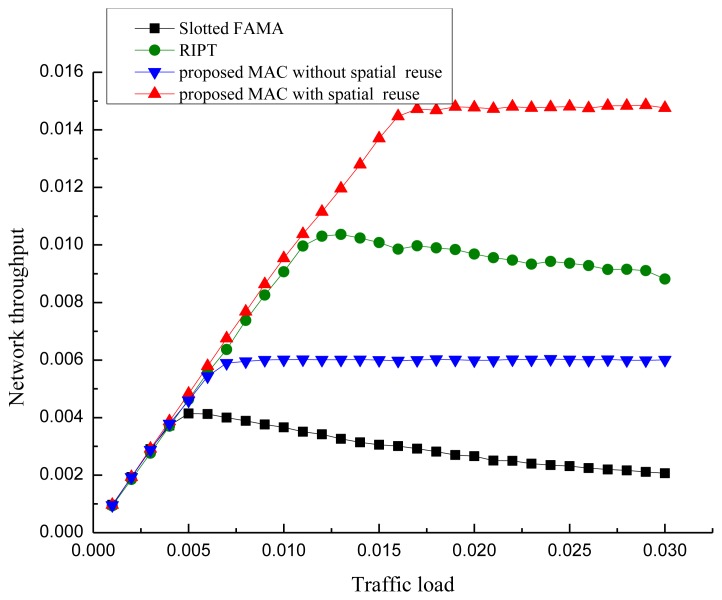
The network throughput.

**Figure 11.**
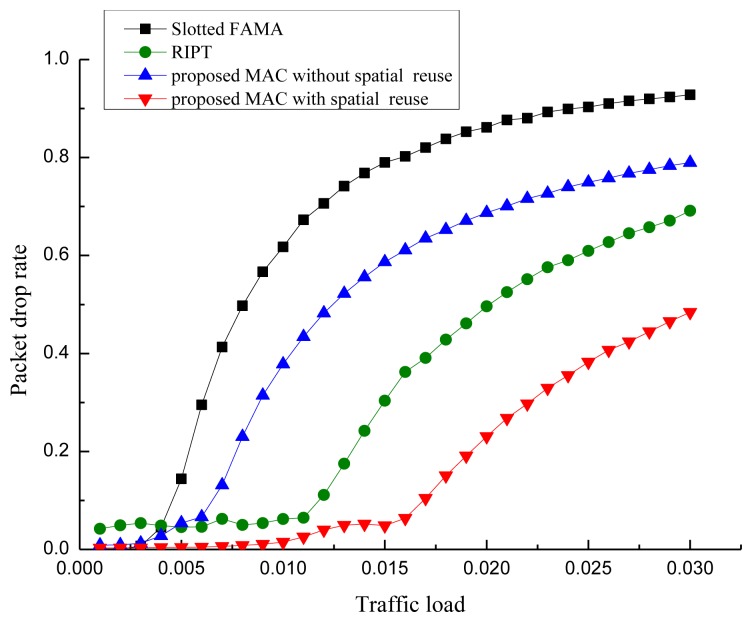
Packet drop rate.

**Figure 12.**
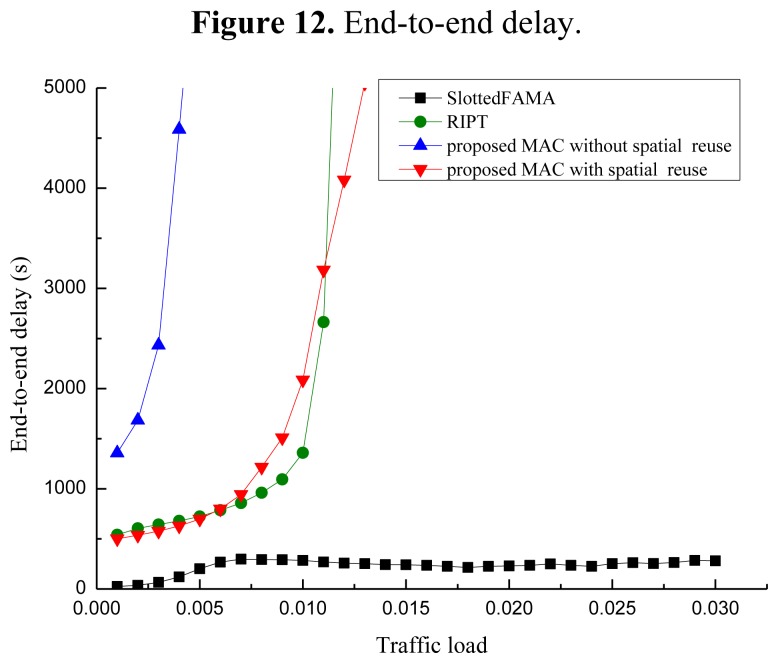
End-to-end delay.

All sensor nodes are equipped with a half-duplex, omni-directional transceiver with a data rate of 1 kbps. The length of control packet is 12.5 bytes. The length of data packet is 500 bytes in the simulations in Figures 10, 11 and 12. The acoustic propagation speed is 1,500 mps. In the RIPT, The average time interval between initiating ready-to-receive (RTRs) at a node (*T*_avg_ in [18]) is 100 s, the number of DATA slots reserved at a receiver (*M*_train_ in [18]) is initialized to 1 and the maximum allowable value for *M*_train_ (*M*_train,max_ in [18]) is 50. In the Slotted FAMA, the *M*_train,max_ is 2 considering that there is only one sender to transmit DATA packet through a handshake. The size of time slot is 4.1 s. In the proposed POCA-CDMA-MAC protocol, the period of the first node starting transmission is the sum of the propagation time and the time for a node to transmit its DATA packet. The length of the spreading sequences for proposed MAC without spatial reuse is 64 considering that there are 16 paths in the networks. The length of the spreading sequences for proposed MAC with spatial reuse is 32 considering that there are only eight one-hop neighbors around the sink. The value of *M*_train,max_ is 2.

Figure 10 shows the performance comparison of the network throughput of the three different MAC protocols, proposed POCA-CDMA-MAC protocol, Slotted FAMA protocol and RIPT MAC protocol. From Figure 10, we observe that the throughput of Slotted FAMA and RIPT MAC protocols increases along with the increase of the traffic load when the traffic load is small, and it decreases slightly along with the increase of the traffic load after reaching peak values. However, the throughput of proposed POCA-CDMA-MAC protocol increases along with the increase of the traffic load when the traffic load is small, and it becomes a relatively stable value when the traffic load increases. The maximum network throughput of Slotted FAMA, the proposed protocol without spatial reuse, RIPT, and the proposed protocol with spatial reuse is 0.004, 0.006, 0.010, and 0.0142 when the traffic load is 0.005, 0.007, 0.013 and 0.018 packet/s, respectively. The achieved maximum network throughput in the network is only about 0.0142, partly because of the long propagation delay and the collision of the data from multiple senders.

From Figure 10, we also observe that the maximum network throughput of the proposed protocol with spatial reuse is the highest among all the protocols.

The reason for this phenomenon is that the packets are transmitted from several paths to the sink without handshaking, while the packets are transmitted with four-way handshaking in the other two protocols. The maximum network throughput of Slotted FAMA protocol is the lowest because that the sink can receive packets from only one neighbor for each round of handshakes in this protocol, while the sink can receive packets chain in the other two MAC protocols. Moreover, we also observe that the maximum network throughput of the proposed protocol without spatial reuse is higher than that of the Slotted FAMA, but it is lower than that of the RIPT protocol. This is because the spreading gain is too large to reduce the channel efficiency. When the spreading gain is reduced in the proposed protocol with spatial reuse, the network throughput is improved.

The comparison of the packet drop rate of the three MAC protocols is shown in Figure 11. From Figure 11, we observe that the packet drop rate of the MAC protocols increases along with the increase of the traffic load. The packet drop rate of the proposed protocol with spatial reuse is much smaller than that of other two protocols when the traffic load is small. This is because the CDMA technology used in the proposed protocol can effectively reduce packet collisions from different paths.

However, the packet drop rate of the proposed protocol without spatial reuse is larger than that of RIPT when the traffic load is larger than 0.05 packet/s. The reason is that the proposed protocol without spatial reuse uses too much time to forward the packets when long spreading sequences are used. In the proposed protocol, the first node is the initiator of the packet transmission, and other nodes must wait for packets from their previous nodes before transmitting their own packets. However, the packet drop rate in RIPT protocol is the largest among the protocols when the traffic load is less than 0.04 packet/s because the receiver-initiated approach used in this protocol makes senders wait too long to send packets.

Figure 12 shows the performance of the end-to-end delay of the three MAC protocols. From Figure 12, we observe that the end-to-end delay increases when the traffic load increases for all three MAC protocols. Among these protocols, the end-to-end delay of Slotted FAMA is the smallest. The reason is that Slotted FAMA is a sender-initiated protocol, and a few packets are forwarded during a handshake round. The end-to-end delay performance of the proposed protocol without spatial reuse is the worst because the packets pre-processed with long spreading sequences need much time to be forwarded.

Moreover, the end-to-end delay of the proposed protocol with spatial reuse is almost as same as that of the RIPT protocol. Hence, the end-to-end delay becomes small when the spreading sequences with relatively short length are used in the proposed protocol with spatial reuse. However, the end-to-end delay of RIPT protocol increases faster than that of the proposed protocol with spatial reuse when the traffic load increases. The reason for this phenomenon is that a sender cannot transmit its DATA packets until a handshake is initiated by the receiver in this protocol.

**Figure 13.**
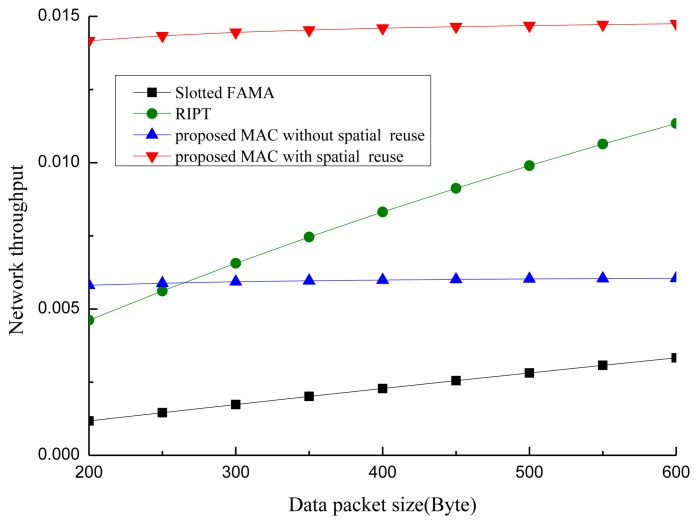
The impact of the data packet size on the network throughput.

Figure 13 shows the impact of the data packet size on the network throughput in three MAC protocols, where the traffic load is 0.018 packet/s. From Figure 13, we observe that the network throughput increases when the data packet size increases. The reason is that the data transmitted in each successful period increases along with the increase of the data packet size. The network throughput of the proposed protocol increases slightly when the data packet size increases since the time for transmitting data packets increases when the data packet size increases.

The network throughput of Slotted FAMA and RIPT increases fast when the data packet size increases. This is because that the rate of the data transmission in each time slot increases as the data packet size increases in these two protocols.

## 5. Discussion

The primary aim of the proposed POCA-CDMA-MAC protocol is to alleviate the funneling effect around the sink in a sink-oriented UWASN. Different from the CDMA technology used in the PLAN protocol [20] and the CDMA-based protocol [21], a round-robin method is used in the proposed protocol, each node, therefore, can transmit its data packets without a handshake mechanism, which reduces the packet collision. Moreover, the POCA is used to reduce the length of the spreading sequences, which improves the channel efficiency.

The round-robin method is used first in the Ordered CSMA MAC protocol [5]. The authors also briefly claimed that the Ordered CSMA works well in a cluster and nodes in different clusters could transmit packets without interference with CDMA technology.

However, there are three significant differences between two protocols. First, the round-robin method is used among nodes within a cluster in the Ordered CSMA protocol, and a node senses the channel and transmits its own data packets in its order, and it does not need receiving and forwarding the data packets from its front node. In the POCA-CDMA-MAC protocol, the round-robin method is used in multi-hop networks, and a node does not transmit its own packets until it has received and forwarded the data packets from its front node. Second, the CDMA technology is suggested to be used between different clusters in the Ordered CSMA protocol, while the CDMA technology is used to different established paths in proposed POCA-CDMA-MAC protocol. Third, a simple, sink-oriented packet transmission is used for avoiding the hidden terminal problem in the multi-hop networks in the POCA-CDMA-MAC protocol, while the Ordered CSMA protocol is designed for a single-hop network.

The acknowledgement of the successful DATA reception is important for the protocol, especially in UWASNs with unreliable channels. In the Slotted FAMA protocol [17], a receiver replies an ACK packet when it has received DATA packet correctly. In the Ordered CSMA MAC protocol [5], a receiver just combines the ACK and DATA packet in one transmission, so the sender waits for a long time to receive the ACK packet. In the proposed protocol, a receiver forwards the DATA packet when it has received it correctly from the sender. Hence, the sender can just wait for to sense its DATA packet forwarded by the receiver. If the sender does not sense its DATA packet forwarded by the receiver, it triggers packet retransmission at the next round. The packet forward mechanism in our proposed MAC protocol saves the energy consumption of sensor nodes without acknowledgements, and makes the protocol work well in multi-hop networks.

However, in order to prevent the sender from listening to a large packet, the receiver can also transmit a small ACK before forwarding the packet from the sender. Then the sender can listen to the small ACK to confirm whether its data packet is received by next-hop node or not. Moreover, when a node near the sink becomes invalid, the sink will assign another node to replace the invalid node. The paths will be re-built and the spreading sequences will be re-assigned. However, the hops of a path around the sink will not be long and the sink will monitor and control the paths, and the nodes far from the sink can random access the channel since the DATA packets transmitted by nodes far from the sink are much less than that transmitted by nodes near the sink.

## 6. Conclusions

In this paper, an improved CDMA-based MAC protocol with a round-robin method and CDMA technology is proposed for UWASNs. In the proposed MAC protocol, each node pre-processes its data packets with a spreading sequence so that the sink can receive data packets from multiple paths at the same time. Moreover, the nodes in the same path are assigned with the same spreading sequence, and they transmit their data packets pre-processed with the assigned spreading sequence in a round-robin method. Hence, the packet collision is reduced and the length of the spreading sequence codes is shortened. Simulation results show that the maximum network throughput of proposed MAC protocol with spatial reuse is almost two times of that of other two protocols, Slotted FAMA protocol and RIPT MAC protocol. Moreover, the proposed MAC protocol also achieves a lower packet drop rate and a smaller end-to-end delay compared to other two MAC protocols.

Since the primary aim of proposed MAC protocol is to alleviate the funneling effect in multi-hop UWASNs, the principle of the protocol is paid attention to rather than the theoretical algorithm. Our future work will concentrate on the development of a proper spread-spectrum code assignment algorithm, the time selection for the first node to send packets, and methods dealing with nodes without data in a round-robin cycle.
